# Effectiveness of travel restrictions in the rapid containment of human influenza: a systematic review

**DOI:** 10.2471/BLT.14.135590

**Published:** 2014-09-29

**Authors:** Ana LP Mateus, Harmony E Otete, Charles R Beck, Gayle P Dolan, Jonathan S Nguyen-Van-Tam

**Affiliations:** a Field Epidemiology Training Programme, Public Health England (East Midlands Office), Nottingham, England.; b University of Nottingham Health Protection and Influenza Research Group, Clinical Sciences Building, Nottingham City Hospital, Hucknall Road, Nottingham, NG5 1PB, England.; c Field Epidemiology Service, Public Health England (North East Office), Newcastle upon Tyne, England.; Correspondence to Jonathan S Nguyen-Van-Tam (email: jvt@nottingham.ac.uk).

## Abstract

**Objective:**

To assess the effectiveness of internal and international travel restrictions in the rapid containment of influenza.

**Methods:**

We conducted a systematic review according to the requirements of the Preferred Reporting Items for Systematic Reviews and Meta-Analyses statement. Health-care databases and grey literature were searched and screened for records published before May 2014. Data extraction and assessments of risk of bias were undertaken by two researchers independently. Results were synthesized in a narrative form.

**Findings:**

The overall risk of bias in the 23 included studies was low to moderate. Internal travel restrictions and international border restrictions delayed the spread of influenza epidemics by one week and two months, respectively. International travel restrictions delayed the spread and peak of epidemics by periods varying between a few days and four months. Travel restrictions reduced the incidence of new cases by less than 3%. Impact was reduced when restrictions were implemented more than six weeks after the notification of epidemics or when the level of transmissibility was high. Travel restrictions would have minimal impact in urban centres with dense populations and travel networks. We found no evidence that travel restrictions would contain influenza within a defined geographical area.

**Conclusion:**

Extensive travel restrictions may delay the dissemination of influenza but cannot prevent it. The evidence does not support travel restrictions as an isolated intervention for the rapid containment of influenza. Travel restrictions would make an extremely limited contribution to any policy for rapid containment of influenza at source during the first emergence of a pandemic virus.

## Résumé

**Objectif:**

Évaluer l'efficacité des mesures de restriction des déplacements internes et internationaux dans le confinement rapide de la grippe.

**Méthodes:**

Nous avons effectué une revue systématique selon les exigences de l'énoncé des items préférables pour rendre compte des revues systématiques ou des méta-analyses (PRISMA). Nous avons effectué des recherches dans les bases de données sur les soins de la santé et la littérature grise et nous avons passé au crible les documents publiés avant mai 2014. L'extraction des données et les évaluations du risque de partialité ont été effectuées par deux chercheurs de manière indépendante. Nous avons fait la synthèse des résultats sous forme narrative.

**Résultats:**

Le risque global de partialité dans les 23 études incluses était faible à modéré. Les mesures de restrictions des déplacements internes et les mesures de restriction aux frontières internationales ont retardé la propagation des épidémies de grippe d'une semaine et de deux mois, respectivement. Les mesures de restriction des déplacements internationaux ont retardé la propagation et le pic de l'épidémie de périodes variant de quelques jours à quatre mois. Les mesures de restriction des déplacements ont réduit de moins de 3% l'incidence des nouveaux cas. L'impact était réduit lorsque des mesures de restriction ont été mises en œuvre plus de six semaines après la notification de l'épidémie ou lorsque le niveau de transmissibilité était élevé. L'impact des mesures de restriction des déplacements serait minime dans les centres urbains où il existe une population dense et des réseaux de transport. Nous n'avons trouvé aucune preuve que les restrictions de déplacement confineraient la grippe dans une zone géographique définie.

**Conclusion:**

Les mesures étendues de restriction des déplacements peuvent retarder la propagation de la grippe, mais ne peuvent pas l'empêcher. Les données probantes n'étayent pas les restrictions de déplacement en tant qu'intervention isolée pour le confinement rapide de la grippe. Les restrictions de déplacement n'apporteraient qu'une contribution extrêmement limitée à toute politique de confinement rapide de la grippe à la source lors de la première apparition d'un virus pandémique.

## Resumen

**Objetivo:**

Evaluar la eficacia de las restricciones a los viajes internos e internacionales en la contención rápida de la gripe.

**Métodos:**

Se realizó una revisión sistemática de acuerdo con la declaración de los requisitos de los elementos de información preferidos para revisiones sistemáticas y meta-análisis. Se examinaron y se realizaron búsquedas de los registros publicados antes de mayo de 2014 en las bases de datos de asistencia sanitaria y en la literatura gris. Dos investigadores llevaron a cabo la extracción de datos y las evaluaciones de riesgo de sesgo de forma independiente. Los resultados se resumieron de forma narrativa.

**Resultados:**

El riesgo general de sesgo en los 23 estudios seleccionados fue de bajo a moderado. Las restricciones a los viajes internos y las restricciones fronterizas internacionales retrasaron la propagación de las epidemias de gripe, al menos una semana y dos meses, respectivamente. Las restricciones a los viajes internacionales retrasaron la difusión, así como el pico de la epidemia por periodos que oscilan entre unos pocos días y cuatro meses. Las restricciones de viajes redujeron la incidencia de casos nuevos a menos del 3%. El efecto se redujo cuando estas restricciones se aplicaron más de seis semanas después de la notificación de epidemias o cuando el nivel de transmisibilidad era alto. El efecto de las restricciones a los viajes sería mínimo en los centros urbanos con poblaciones de alta densidad y redes de viaje. No se encontraron pruebas de que las restricciones a los viajes podrían contener la gripe en un área geográfica definida.

**Conclusión:**

Las restricciones amplias a los viajes pueden retrasar la difusión de la gripe, si bien no pueden prevenirla. Las pruebas no apoyan las restricciones a los viajes como una intervención aislada para la contención rápida de la gripe. Las restricciones a los viajes podrían contribuir de forma muy limitada a una política de contención rápida de la gripe en origen durante la primera aparición de un virus pandémico.

## ملخص

**الغرض:**

تقييم فعالية القيود على السفر الداخلي والدولي في الاحتواء السريع للأنفلونزا.

**الطريقة:**

قمنا بإجراء استعراض منهجي وفقاً لمتطلبات البنود المتعلقة بتقديم التقارير المفضلة لبيان الاستعراضات المنهجية والتحليلات الوصفية. وتم البحث في قواعد بيانات الرعاية الصحية والمؤلفات غير الرسمية وفحصها لمعرفة السجلات التي تم نشرها قبل أيار/مايو 2014. وأجرى باحثان استخلاص البيانات وتقييمات خطورة التحيز بشكل مستقل. وتم تجميع النتائج بشكل سردي.

**النتائج:**

تراوحت خطورة التحيز بشكل عام في الدراسات المدرجة البالغ عددها 23 دراسة من منخفضة إلى متوسطة. وأدت القيود المفروضة على السفر الداخلي والقيود المفروضة على الحدود الدولية إلى تأخير انتشار أوبئة الأنفلونزا بأسبوع واحد وشهرين، على التوالي. وأدت القيود المفروضة على السفر الدولي إلى تأخير انتشار الأوبئة وذروتها بفترات تتراوح بين بضعة أيام وأربعة أشهر. وأدت القيود على السفر إلى تقليل الإصابة بالحالات الجديدة بأقل من 3 %. وانخفض الأثر عند تنفيذ القيود بعد الإبلاغ عن الأوبئة بأكثر من ستة أسابيع وعند ارتفاع مستوى قابلية السريان. وكان أقل أثر للقيود على السفر في المراكز الحضرية ذات الكثافة السكانية وشبكات السفر. ولم نعثر على بيّنات تفيد بأن القيود المفروضة على السفر يمكنها احتواء الأنفلونزا داخل منطقة جغرافية معينة.

**الاستنتاج:**

يحتمل أن تؤدي القيود الموسعة على السفر إلى تأخير انتشار الأنفلونزا غير أنه لا يمكنها توقيه. ولا تدعم البيّنات القيود على السفر كتدخل فردي بهدف الاحتواء السريع للأنفلونزا. ومن الممكن أن تسهم القيود المفروضة على السفر بشكل محدود للغاية في أي سياسة تهدف إلى الاحتواء السريع للأنفلونزا عند مصدرها خلال الظهور الأول لفيروس الجائحة.

## 摘要

**目的:**

评估国内和国际出行限制对快速控制流感的有效性。

**方法:**

我们根据系统回顾和荟萃分析首选报告项目的需求进行了一项系统回顾。搜索医疗数据库和灰色文献并筛选在2014年5月前发表的记录。由两位研究者独立执行数据提取和误差风险评估。以叙事形式综合结果。

**结果:**

在纳入的23项研究中,整体误差风险为中低等级。国内出行限制和国境线限制分别将流感流行传播推迟一个星期和两个月。国际出行限制将流行病传播和高峰期延迟几天到四个月不等。出行限制减少的新病例发病率不到3%。流行病通知发布超过六周后或在传播等级较高时,实施限制措施的影响效果趋于减少。出行限制对具有密集人口和出行网络的城市中心影响最小。我们没有发现旅游限制将流感控制在某一特定地理区域的证据。

**结论:**

广泛的出行限制可能会推迟流感的传播,但没有阻止作用。证据不支持出行限制是一个快速控制流感的独立干预。对于任何要在大流行性流感病毒刚刚出现时就从源头快速控制流感的政策来说,出行限制的作用非常有限。

## Резюме

**Цель:**

Оценить эффективность ограничений на внутренние и международные поездки в целях предотвращения быстрого распространения гриппа.

**Методы:**

Был проведен систематический обзор в соответствии с рекомендациями о наиболее предпочтительных параметрах отчетности для систематических обзоров и мета-анализа. Поиск и отбор соответствующей информации был осуществлен в медицинских базах данных и неиндексированной литературе, опубликованной до мая 2014 г. Отбор данных и оценка риска систематической ошибки проводились двумя исследователями независимо друг от друга. Результаты были обобщены в форме отчета.

**Результаты:**

Общий риск систематической ошибки в 23 включенных исследованиях был низким или умеренным. Ограничения на внутренние поездки и на пересечение международных границ задерживали распространение эпидемий гриппа на одну неделю и два месяца соответственно. Ограничения на международные поездки задерживали распространение и пик эпидемий на период от нескольких дней до четырех месяцев. Ограничения на поездки сокращали число новых случаев менее чем на 3%. Эффект снижался, если меры по ограничению поездок принимались по истечении шести месяцев после уведомления об эпидемиях или когда уровень переносимости заболевания был уже высоким. Ограничения на поездки оказывали минимальное влияние в городских центрах с высокой плотностью населения и разветленной сетью пассажирских перевозок. Доказательства того, что ограничения на поездки препятствуют распространению гриппа за пределы определенного географического региона не найдены.

**Вывод:**

Масштабные меры по ограничению поездок могут замедлить распространение гриппа, но не могут предотвратить его. Факты, подтверждающие, что ограничения на поездки, как отдельная мера, предотвращают быстрое распространение гриппа, не найдены. Ограничения на поездки в чрезвычайно малой степени способствуют быстрой локализации гриппа в месте его возникновения при первом появлении пандемического вируса.

## Introduction

Travel restrictions were included in the *WHO interim protocol: rapid operations to contain the initial emergence of pandemic influenza* that was published in 2007 by the World Health Organization (WHO).^1^ However, as they would hamper global travel and trade, such restrictions are not recommended by WHO once the global spread of pandemic influenza is established.^2^^,^^3^ In 2009, some countries applied travel restrictions as one of several strategies to prevent the introduction of the influenza virus A(H1N1)pdm09 into their territories but the effectiveness of this approach has subsequently been questioned.^4^ Research on influenza has focused on the evaluation of the effectiveness and impact of pharmaceutical interventions.^5^ As quantitative assessment of the effectiveness of travel restrictions in pandemic situations tends to be more challenging, there are scarce data on this topic. In any meta-analysis of surveillance data from multiple studies, it is difficult to quantify and compare the effectiveness of travel restrictions because such interventions are frequently implemented with other countermeasures and without following standardized protocols.^6^ However, mathematical models can be used to predict the effectiveness of each type of intervention and inform policy-makers at national and international levels. In 2009, a systematic review of studies based on such models revealed limited evidence of the effectiveness of restrictions in air travel – within and between countries – in the containment of pandemic influenza.^7^ There has been no more recent systematic assessment of the effectiveness of restrictions in land, sea or air travel as isolated interventions. We therefore decided to assess the effectiveness of travel restrictions in the rapid containment of influenza strains with pandemic potential, in a systematic review that incorporated data collected during the 2009 pandemic.

## Methods

Before commencement, our protocol was registered with PROSPERO – the international prospective register of scientific reviews maintained by the United Kingdom of Great Britain and Northern Ireland’s National Institute for Health Research.^8^ We conducted a systematic review according to the requirements of the Preferred Reporting Items for Systematic Reviews and Meta-Analyses statement.^9^ We assessed the evidence for restrictions in internal travel – travel within the same country – or international travel – travel between two or more countries – affecting the spread of influenza. We considered the air, terrestrial or maritime transportation of humans to or within countries affected by seasonal or pandemic influenza. The outcome measures of interest were epidemiological characteristics and some viral transmission parameters of influenza such as the basic reproductive number (*R*_0_). Studies eligible for inclusion were reports, reviews, meta-analyses, mathematical modelling studies and observational and experimental studies published before May 2014. Studies that only evaluated the spread of influenza in animals or animal products were excluded.

### Search strategy

We searched numerous health-care databases and sources of grey literature (Box 1). Critical keywords and thesaurus heading terms were initially tailored to MEDLINE searches and then adapted for other sources as necessary. The full search construct was included in the registered protocol.^10^ We contacted field experts and undertook reference and citation tracking to identify further relevant literature.

Box 1 **Sources of literature included in this systematic review**

**Health-care databases**

- CINAHL (Cumulative Index to Nursing and Allied Health Literature)
- Cochrane Library – Central Register of Controlled Trials
- EMBASE
- PubMed – including MEDLINE
- World Health Organization Global Index Medicus

**Evidence-based reviews**

- Bandolier
- Cochrane Library – Cochrane Database of Systematic Reviews, Database of Abstracts of Reviews of Effects, Health Technology Assessment Database, NHS Economic Evaluation Database

**Guidelines**

- United Kingdom Department of Health
- United Kingdom National Institute for Health Care and Excellence – Evidence Search
- United States Centers for Disease Control and Prevention – Guidance

**Grey literature**

- Consultation with domain experts – Martin Cetron (Centers for Disease Control and Prevention, Atlanta), John Edmunds (London School of Hygiene & Tropical Medicine, London), Peter Grove (Department of Health, London), Richard J Pitman (Oxford Outcomes, Oxford)
- OpenSIGLE system for information on grey literature in Europe
- United Kingdom National Institute for Health Care and Excellence – Evidence Search
- Web of Science

**Manual searching of relevant journals**

- Eurosurveillance
- Emerging Infectious Diseases

**Reference tracking**

- Reference lists of all studies selected for inclusion were searched to identify further relevant studies

**Citation tracking**

- Web of Science – Science Citation Index
- Google Scholar

**Internet searching**

- www.google.com
- www.dh.gov.uk
- www.hpa.org.uk – now: www.phe.gov
- www.who.int
- www.cdc.gov
- www.flu.gov

### Study selection

All records identified were imported into the EndNote X6 software package (Thomson Reuters, San Francisco, United States of America). Following the removal of duplicates, all remaining records were screened for inclusion against the protocol’s eligibility criteria by two researchers.^8^ We used a three-stage sifting approach to review titles, abstracts and full texts. Where disagreements arose, a third reviewer provided arbitration.^8^

### Data extraction

All records that met the eligibility criteria were subject to data extraction. Two reviewers independently extracted study data using a piloted form; any disagreements were resolved with a third reviewer. The full list of data items extracted is available on PROSPERO.^8^

### Assessing risk of bias

Risk of bias was assessed at both study and outcome level. We used an evaluation tool developed by the United States Agency for Healthcare Research and Quality^11^ for assessing such risk in reviews. Since we are not aware of a previously validated instrument to assess risk of bias in mathematical modelling studies, we developed a tool based on the principles for the construction of mathematical models recommended by the London School of Hygiene & Tropical Medicine,^12^ in consultation with an experienced modeller^8^ (see Appendix A; available at: http://www.nottingham.ac.uk/research/groups/healthprotection/documents/supplementary-data-sr-travel-restrictions-influenza-mateus-et-al-220914.pdf).

### Summary measures and data synthesis

Descriptive statistics were calculated using Excel 2010 (Microsoft, Richmond, USA). We used a recognized framework to synthesize the extracted data and assessments of risk of bias in a narrative style.^13^

## Results

### Study selection and characteristics

**Fig. 1:**
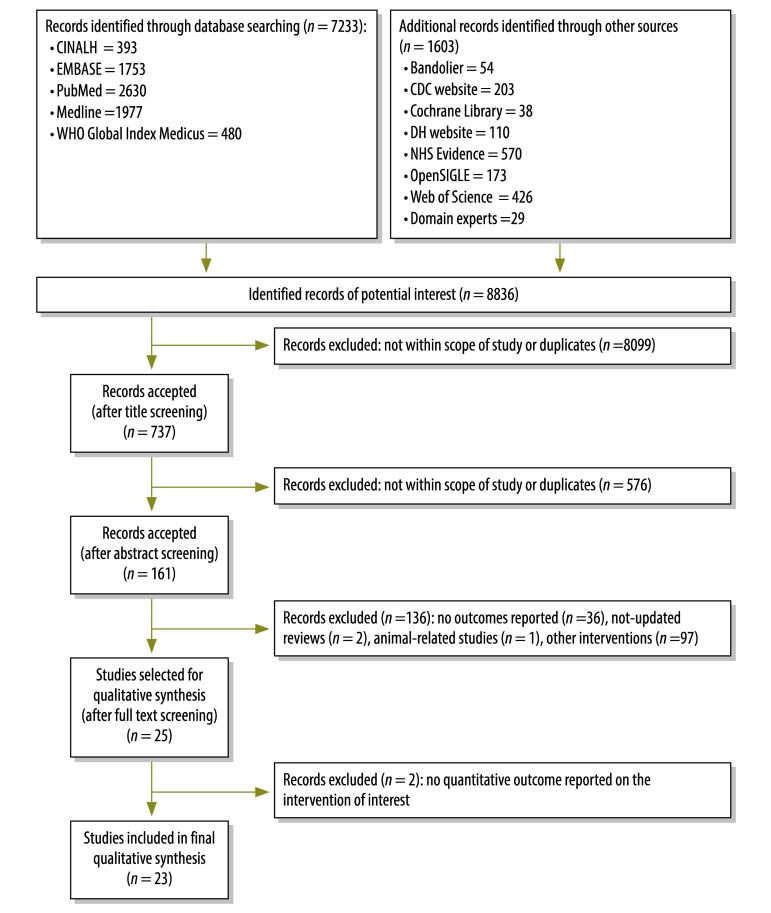
**Flowchart for the selection of studies on the effectiveness of travel restriction in the containment of human influenza**

CDC: United States Centers for Disease Control and Prevention; CINAHL: Cumulative Index to Nursing and Allied Health Literature; DH: United Kingdom Department of Health; NHS: United Kingdom National Health Service; WHO: World Health Organization.

Before removal of duplicates, we identified 8836 potentially relevant records. However, only 23 studies – 19 mathematical modelling studies, one time-series analysis, two literature reviews and one systematic review – met our eligibility criteria (Fig. 1).^4^^,^^7^^,^^14^^–^^34^

Of the modelling studies included, 14 used stochastic models,^4^^,^^15^^,^^16^^,^^22^^,^^23^^,^^25^^–^^29^^,^^31^^–^^34^ two used deterministic models,^18^^,^^19^ two used a combination of both stochastic and deterministic methods^14^^,^^17^ and one used a Poisson regression model.^24^ Six studies^15^^–^^19^^,^^31^ were based on meta-population models of influenza spread^35^ and one^4^ on an alternative model.^36^ The focus of the included studies was the effectiveness of internal^22^^,^^23^^,^^26^^,^^27^^,^^29^ or international^4^^,^^14^^–^^19^^,^^24^^,^^25^^,^^31^^–^^34^ travel restrictions or combined internal and international travel restrictions.^28^^,^^30^ All but three of our included studies involved assessments of the impact of restrictions on air travel.^22^^,^^25^^,^^26^ Only one assessed the impact of restrictions on aerial, maritime and terrestrial transportation.^34^ The characteristics of the included modelling studies and time-series analysis are presented in Appendix A.

The systematic review that we included synthesized evidence from modelling studies published between 1990 and September 2009.^7^ The literature reviews that we included evaluated evidence from mathematical modelling studies on the containment of pandemic influenza and evidence used for preparedness planning in the United Kingdom.^20^^,^^21^

### Risk of bias within studies

Of the 20 studies based on mathematical modelling or time-series analysis, 17 were found to be at low risk of bias (Table 1). The other three were found to be at moderate risk of bias –because of limitations in the study design^22^^,^^24^ or the low quality of travel data.^25^ Methodological issues that may have led to bias included a lack of transmission variation during the progression of epidemics, seasonality, heterogeneous mixing and varying susceptibility of populations.^14^^,^^26^^,^^27^^,^^29^^,^^34^

**Table 1:** Risk of bias assessments of mathematical modelling studies or time-series analysis on the effectiveness of travel restrictions to reduce influenza transmission

Study	Domain of bias^a^											
	Research question(s) precise and clear	Primary findings presented	Original findings	Model techniques or model structure used	Appropriate model complexity	Suitable mathematical modelling	Input data sources identified	Major model assumptions described	Relevant factors explored	Model validated	Techniques used for model fitting	Sensitivity analysis
Bajardi et al. (2011)^4^	Low	Low	Low	Low	Low	Low	Low	Low	Low	Low	Low	Low
Bolton et al. (2012)^26^	Low	Low	Low	Low	Low	Low	Low	Low	Low	Low	Moderate	Low
Brownstein et al. (2006)^30^^,b^	Low	Low	Low	Low	Low	Low	Low	Low	Low	Low	Low	High
Chong and Ying Zee (2012)^34^	Low	Low	Low	Low	Low	Low	Low	Low	Low	NS	Low	Low
Ciofi degli Atti et al. (2008)^17^	Low	Low	Low	Low	Low	Low	Moderate	Low	Low	Low	Low	High
Colizza et al. (2007)^15^	Low	Low	Low	Low	Low	Low	Low	Low	Low	NS	NS	Low
Cooper et al. (2006)^16^	Low	Low	Low	Low	Low	Low	Low	Low	Low	NS	Low	Low
Eichner et al. (2009)^25^	Low	Low	Low	Low	Moderate	Low	Moderate	Low	Low	NS	NS	High
Epstein et al. (2007)^31^	Low	Low	Moderate	Low	Low	Low	Low	Low	Low	NS	NS	Low
Ferguson et al. (2006)^28^	Low	Low	Low	Low	Low	Low	Low	Low	Low	High	Low	Low
Flahault et al. (2006)^18^	Low	Low	Low	Low	Moderate	Low	Moderate	Low	Low	NS	NS	Low
Germann et al. (2006)^27^	Low	Low	Low	Low	Low	Low	Low	Low	Low	High	NS	Low
Hsieh et al. (2007)^22^	Low	Low	Low	Moderate	Low	Moderate	Low	Low	Low	NS	NS	High
Hollingsworth et al. (2006)^33^	Low	Low	Moderate	Low	Low	Low	Moderate	Low	Low	NS	NS	High
Kernéis et al. (2008)^19^	Low	Low	Low	Low	Low	Low	Low	Low	Low	High	Low	Low
Lam et al. (2011)^14^	Low	Low	Low	Low	Low	Low	Moderate	Low	Low	High	No	Low
Lee et al. (2012)^23^	Low	Low	Low	Low	Low	Low	Low	Low	Low	High	Low	Low
Marcelino & Kaiser (2012)^32^	Low	Low	Low	Low	Low	Low	Low	Low	Low	High	NS	Low
Scalia Tomba & Wallinga (2008)^24^	Low	Low	Low	Low	Moderate	Moderate	Moderate	Low	Low	High	NS	High
Wood et al. (2007)^29^	Low	Low	Low	Low	Low	Low	Low	Low	Low	NS	NS	Low

NS: not specified.

^a^ For each domain of interest, risk of bias was categorized as low if the authors addressed the domain adequately, moderate if the authors’ coverage of the domain was superficial or incomplete, and high if the authors reported coverage of the domain was poor.

^b^ As this study contained mainly modelling components relevant to the outcomes, it was assessed for risk of bias as a modelling study.

The systematic and literature reviews were at moderate risk of bias (Table 2). The systematic review^7^ was based on literature from only one health-care database and on a snow-balling strategy that could have introduced selection bias. Neither of the literature reviews included any assessment of the design and quality of the studies that were included or detailed descriptions of the eligibility criteria applied.^20^^,^^21^

**Table 2:** Risk of bias assessments of systematic or literature reviews on the effectiveness of travel restrictions to reduce influenza transmission

Study	Domain of bias^a^										Funding or sponsorship
	Study question(s)	Search strategy	Inclusion and exclusion criteria	Intervention(s)	Outcomes	Data extraction	Study quality and validity	Data synthesis and evaluation	Results	Discussion	
Department of Health (2011)^20^	Low	Low	Moderate	Low	Low	High	Moderate	Low	Low	Low	UKDH
Department of Health (2012)^21^	Low	High	Moderate	Low	Low	High	High	Low	Low	Low	UKDH
Lee et al. (2009)^7^	Low	Low	Low	Low	Low	Low	Moderate	Low	Low	Low	NS

NS: not specified; UKDH: United Kingdom of Great Britain and Northern Ireland Department of Health.

^a^ For each domain of interest, risk of bias was categorized as low if the authors addressed the domain adequately, moderate if the authors’ coverage of the domain was superficial or incomplete, and high if the authors reported coverage of the domain was poor.

### Synthesis of results

#### Internal travel restrictions

Travel restrictions appeared to have limited effectiveness in the containment of influenza at local level (Table 3 and Table 4; Table 3 is available at: http://www.who.int/bulletin/volumes/92/12/14-135590). 

**Table 3:** Simulated effects of the implementation of internal travel restrictions on the spread and duration of pandemic or epidemic influenza

Study	Type of restrictions and setting	Study design	Influenza strain involved	Strain transmissibility (*R*_0_)	Scenario and duration interventions	Effect estimate
Bolton et al. (2012)^26^	Internal road and rail, Mongolia	Mathematical stochastic model^a^	Pandemic influenza A H1N1 pdm09	1.6	50% travel restriction, 2 weeks	Pandemic peak delayed 1 week
					50% travel restriction, 4 weeks	Pandemic peak delayed 1.5 weeks
Brownstein et al. (2006)^30^	Internal and international air, USA	Time-series analysis	Seasonal influenza	1.4, 1.7 or 2.0	Travel restricted to and from a city with > 1000 infectious cases or worldwide when > 1000 such cases in city of origin, the 2001–2002 influenza season	Peak mortality due to influenza delayed 16 days
Department of Health (2012)^21^	Several scenarios	Literature review (mathematical models)	Pandemic influenza	NS	90% internal travel restriction between localities	Little effect on the length of epidemic and size of peak in each local area
					90% internal travel restriction between localities plus total ban on international flights	Increased spread of national epidemics and desynchronization of epidemics in local areas
Ferguson et al. (2006)^28^	Internal air, plus border controls, England, Scotland and Wales in United Kingdom and USA	Mathematical stochastic model^b^	Novel pandemic influenza strain	1.4–2.0	Internal travel restriction – implemented when 50 cases reported in affected country – plus 99%-effective border restrictions stopping entry of infected travellers – implemented from day 30 of global pandemic	ES delayed 2–3 weeks in USA but not delayed in United Kingdom^c^
				1.4–2.0	Internal travel restriction in USA	ES delayed 1 week in USA but not delayed in United Kingdom^d^
				1.4–2.0	75% internal travel restriction – i.e. blanket or reactive movement restrictions^e^	No impact on ES
				1.7 or 2.0	USA only: border restrictions plus closure of all airports in USA to internal flights	With *R*_0_ set to 1.7 or 2.0, EP delayed 49 days
					USA only: border restrictions plus reactive movement restrictions with 20-km exclusion zone	With *R*_0_ set to 1.7 or 2.0, EP delayed 54 days
					USA only: border restrictions but no blanket movement restrictions	With *R*_0_ set to 1.7, EP delayed 60 days
					USA only: border restrictions plus 50-km blanket movement restrictions	With *R*_0_ set to 1.7 or 2.0, EP delayed 44 days
					USA only: reactive movement restrictions with 20-km exclusion zone	With *R*_0_ set to 1.7 or 2.0, EP delayed 6 days
					USA only: border restrictions plus 20-km blanket movement restrictions	With *R*_0_ set to 2.0, EP delayed 60 days
Germann et al. (2006)^27^	Internal, USA	Mathematical stochastic model^b^	H5N1 pandemic influenza	1.6, 1.9. 2.1 or 2.4	90% reduction in long-distance domestic travel when 10 000 symptomatic individuals have been recorded in USA, 180 days	EP delayed by a few days – when *R*_0_ is relatively high – to a few weeks
Lee et al. (2012)^23^	Restrictions on internal migration, restrictions by airplane, car, bus or ship, Republic of Korea	Mathematical stochastic single-city and multi-city extended models^b^	Human influenza	1.0, 1.2, 1.5 or 1.8	50% travel restriction, similar parameters all cities, constant infection force	Slight – unspecified – delay in EP. Size of EP reduced by < 0.01%
					> 90% travel restriction, similar parameters all cities, variation in infection force	Unspecified delay in EP. Delayed spread of epidemic into new cities but increased risk of localized larger outbreaks
Lee et al. (2009)^7^	Several scenarios	Systematic review (deterministic and stochastic models)	Different strains of pandemic influenza	1.7–2.0	Internal and international air travel restriction	ES delayed 2–3 weeks if restrictions 99% effective
Wood et al. (2007)^29^	Internal, Australia	Mathematical stochastic model^f^	Pandemic influenza	1.5, 2.5 or 3.5	80% restriction of travel from Sydney to Melbourne, variable infectivity, 2 weeks after epidemic	With *R*_0_ set to 1.5, ES delayed a median of 32 days
					As above except constant infectivity	With *R*_0_ set to 1.5, 2.5 and 3.5, ES delayed a median of 30, 22 and 16 days, respectively
					As above except peak infectivity	With *R*_0_ set to 1.5, 2.5 and 3.5, ES delayed a median of 22, 15 and 11 days, respectively
					80% restriction of travel from Darwin to Sydney, constant infectivity, 2 weeks after epidemic	With *R*_0_ set to 1.5, 2.5 and 3.5, ES delayed a median of 34, 17 and 13 days, respectively
					As above except peak infectivity	With *R*_0_ set to 1.5, 2.5 and 3.5, ES delayed a median of 24, 12 and 9 days, respectively
					80% travel restriction nationwide, 4 weeks after epidemic began	No impact with *R*_0_ set to 1.5
					90% restriction of travel from Sydney to Melbourne, constant infectivity, 2 weeks after epidemic began	With *R*_0_ set to 1.5, 2.5 and 3.5, ES delayed a median of 53, 25 and 18 days, respectively
					As above except peak infectivity	With *R*_0_ set to 1.5, 2.5 and 3.5, ES delayed a median of 32, 17 and 13 days, respectively
					90% restriction of travel from Darwin to Sydney, constant infectivity, 2 weeks after epidemic began	With *R*_0_ set to 1.5, 2.5 and 3.5, ES delayed a median of 41, 20 and 15 days, respectively
					As above except peak infectivity	With *R*_0_ set to 1.5, 2.5 and 3.5, ES delayed a median of 25, 14 and 10 days, respectively
					99% restriction of travel from Sydney to Melbourne, constant infectivity, 2 weeks after epidemic began	With *R*_0_ set to 1.5, 2.5 and 3.5, ES delayed a median of 75, 34 and 25 days, respectively
					As above except peak infectivity	With *R*_0_ set to 1.5, 2.5 and 3.5, ES delayed a median of 52, 24 and 17 days, respectively
					99% restriction of travel from Darwin to Sydney, constant infectivity, 2 weeks after epidemic began	With *R*_0_ set to 1.5, 2.5 and 3.5, ES delayed a median of 75, 30 and 22 days, respectively
					As above except peak infectivity	With *R*_0_ set to 1.5, 2.5 and 3.5, ES delayed a median of 46, 21 and 15 days, respectively

EP: epidemic peak; ES: epidemic spread; NS: not specified; *R*_0_: basic reproductive number.

^a^ A so-called SEIAR model, in which individuals who are susceptible (S), exposed (E), infectious and presented for medical care (I), infectious but not presented for medical care (A) or recovered (R) are considered.

^b^ A so-called SEIR model, in which individuals who are susceptible (S), exposed (E), infectious (I) or recovered (R) are considered.

^c^ Internal travel restrictions only effective if implemented within 2 weeks of first case in the USA. Border controls only effective if they prevent entrance of 99% of infective travellers and are implemented within 45 days of the start of pandemic.

^d^ Internal travel restrictions only effective if implemented within 2 weeks of first case in the USA.

^e^ With reactive movement restrictions, a 20-km exclusion zone is established around every diagnosed case – with merging of overlapping zones – and movement in and out of each exclusion zone is eliminated. With blanket movement restrictions, all journeys by an individual from that individual’s home that exceed a certain distance – often 20 or 50 km – are eliminated.

^f^ A so-called SIR model, in which individuals who are susceptible (S), infected (I) or recovered (R) are considered.

**Table 4:** Simulated impact of internal travel restrictions on influenza and influenza-like illness in influenza pandemics or epidemics

Study	Type of restrictions and setting	Study design	Influenza strain involved	Strain transmissibility (*R*_0_)	Scenario and duration of intervention	Effect estimate
Bolton et al. (2012)^26^	Internal road and rail, Mongolia	Mathematical stochastic model^a^	Pandemic influenza A H1N1 pdm09	1.6	95% travel restriction, 2–4 weeks	12% reduction in ILI peak and a reduction in mean attack rate of < 0.1%, even when restrictions with 95% effectiveness are implemented for 4 weeks
Ferguson et al. (2006)^28^	Internal air, plus border controls, England, Scotland, and Wales in United Kingdom and USA	Mathematical stochastic model^b^	Novel pandemic influenza strain	1.4–2.0	Internal travel restrictions – i.e. blanket or reactive movement restrictions^c^ – at 90–100% levels of effectiveness	Reduction in attack rate of < 2%
Germann et al. (2006)^27^	Internal, USA	Stochastic single- city and multi-city extended models^d^	H5N1 pandemic influenza	1.6, 1.9, 2.1 or 2.4	90% reduction in long-distance domestic travel when 10 000 symptomatic individuals have been recorded in USA, 180 days	With *R*_0_ set to 1.6, 1.9, 2.1 and 2.4, cumulative incidence per 100 inhabitants was 32.8 (32.6), 44.0 (43.5), 48.9 (48.5) and 54.1 (53.7) cases, respectively^e^
Hsieh et al. (2007)^22^	Internal, China	Mathematical stochastic patch model^d^	Human seasonal influenza	NS	Travel of symptomatic individuals from areas of low prevalence to areas of high prevalence eliminated	Decreased *R*_0_ to < 1, preventing spread of epidemic
					Travel of symptomatic individuals from areas of high prevalence to areas of low prevalence eliminated	Increased R_0_ to > 1, prolonging the epidemic

ILI: influenza-like illness; NS: not specified; *R*_0_: basic reproductive number.

^a^ A so-called SEIAR model, in which individuals who are susceptible (S), exposed (E), infectious and presented for medical care (I), infectious but not presented for medical care (A) or recovered (R) are considered.

^b^ A so-called SEIR model, in which individuals who are susceptible (S), exposed (E), infectious (I) or recovered (R) are considered.

^c^ With reactive movement restrictions, a 20-km exclusion zone is established around every diagnosed case – with merging of overlapping zones – and movement in and out of each exclusion zone is eliminated. With blanket movement restrictions, all journeys by an individual from that individual’s home that exceed a certain distance – often 20 or 50 km – are eliminated.

^d^ A so-called SEIRP model, in which individuals who are susceptible (S), incubating (E), infective (I), recovered (R) or partially immune (P) are considered.

^e^ The values in parentheses indicate the cumulative incidences seen – in the corresponding baseline scenarios – with no interventions.

With pandemic influenza A(H1N1)pdm09 in Mongolia, the estimated delay of the pandemic peak varied between 1.0 and 1.5 weeks when 50% road and rail travel restrictions over 2–4 weeks were simulated.^26^ The corresponding impact on the attack rate was minimal – e.g. 95% travel restrictions led to a reduction of just 0.1%.^26^ A study set in the USA revealed similar findings – e.g. a delay in spread of 2–3 weeks if travel restrictions were 99% effective and implemented in conjunction with border restrictions that prevented the entry of infected travellers.^28^ Travel restrictions alone could delay spread by 1 week but only if implemented within 2 weeks of the first case.^28^ In one simulation, border controls preventing 99.9% of cases entering any given country delayed epidemic spread by up to 35 days.^24^ Another study in the USA presented analogous results – e.g. a 90% restriction on long-distance flights led to delays in the epidemic peak that ranged between a few days and a few weeks.^27^ Effectiveness of travel restrictions decreased as the transmissibility of the strain increased; travel restrictions reduced the incidence of new cases by less than 3%.^27^ According to a time-series analysis in the USA, a 50% restriction in air travel during the 2001–2002 influenza season would have delayed the peak mortality associated with novel strains of seasonal influenza by 16 days – i.e. compared with the timing of the peak in previous years.^30^

Internal travel restrictions in England, Scotland and Wales in the United Kingdom were predicted to have minimal impact on the magnitude of the peak and in delaying the spread of the epidemic – possibly because there are some densely populated urban areas and relatively high levels of population movement.^28^ However, in a recent review, it was estimated that a combination of internal and international travel restrictions could help to stagger the impact of a pandemic within a country such as the United Kingdom, by desynchronizing localized outbreaks.^21^ In Australia, it was reported that the impact of 80–99% restriction of air travel between major city hubs was less when varying transmissibility rather than constant transmissibility was simulated. ^29^ In the same investigation, effectiveness fell when strain transmissibility was increased.^29^ In the Republic of Korea, restriction of travel between cities by more than 50% reduced the epidemic peak by less than 0.01% when constant transmissibility was modelled.^23^ When variations in transmissibility were simulated, such travel had to be restricted by more than 90% for the epidemic peak to be delayed significantly.^23^ Travel restrictions would reduce the spread to new cities but could also increase the risk of large localized outbreaks.^23^ In China, it was observed that overall *R*_0_ would increase if symptomatic travellers were banned from moving from areas with high prevalence of seasonal influenza to areas with low prevalence. When symptomatic travellers were banned from leaving low-prevalence areas, a decrease in overall R_0_ to less than one was predicted.^22^

#### International travel restrictions

International travel restrictions also appeared to have limited effectiveness (Table 5 and Table 6; Table 6 is available at: http://www.who.int/bulletin/volumes/92/12/14-135590). Low-level restrictions – i.e. restrictions of less than 70% – were the least effective in containing the spread of epidemics between countries. It was found that a 40% restriction of air travel would only delay the spread of influenza A(H1N1)pdm09 from Mexico to other countries by less than 3 days.^4^ In a high transmissibility scenario, a 20% or even a 50% reduction in the volume of travellers would not have any significant impact on the global spread of influenza A(H5N1).^15^ In a meta-population model of pandemic influenza, based on the 1968–1969 influenza A(H3N2) pandemic virus it was predicted delays in the epidemic peak of 9 and 14 days with 50% and 90% restriction of air travel, respectively.^18^

**Table 5:** Simulated effects of the implementation of international travel restrictions on the spread and duration of pandemic or epidemic influenza

Study	Type of restrictions and setting	Study design	Influenza strain involved	Strain transmissibility (*R*_0_)	Scenario and duration of intervention	Effect estimate
Bajardi et al. (2011)^4^	Air travel, global	Mathematical stochastic model^a^	A(H1N1)pdm09 epidemic	NS	40% restriction, < 6 weeks from epidemic notification	ES to other countries delayed < 3 days
					90% restriction, < 6 weeks from epidemic notification	ES to other countries delayed < 2 weeks
					Any level of restriction, > 6 weeks from epidemic notification	No impact
Brownstein et al. (2006)^30^	Internal and international air travel, USA	Time-series analysis	Seasonal influenza	1.4, 1.7 or 2.0	Travel restricted to and from a city with > 1000 infectious cases or worldwide when > 1000 such cases in city of origin, the 2001–2002 influenza season	Seasonal influenza season prolonged by 16 days
Chong and Ying Zee (2012)^34^	Air, sea and land travel, Hong Kong Special Administrative Region, China	Mathematical stochastic model^a^	A(H1N1) pdm09	1.1	99% air, land and sea travel	EP delayed up to 1 year
				1.4	90% air, land and sea	ES and EP delayed 4 and 6 weeks, respectively
					99% air, land and sea	ES and EP delayed 2 and 3 months, respectively
					99% air and land	ES and EP delayed 1–2 and 3.5 weeks, respectively
					99% air	EP delayed up to 2 weeks
					99% land	EP delayed up to 1 week
					99% sea	EP delayed up to 1 week
				1.7	90% air, land and sea	No significant impact on timing of EP
					99% air, land and sea	EP delayed up to 8 weeks
Ciofi degli Atti et al. (2008)^17^	Air travel, Italy	Mathematical global determinist model^a^	A(H5N1)	1.4, 1.7 or 2.0	90% air travel restriction, implemented 30 days after first case in pandemic was recorded or < 2 months after the introduction of first case in Italy	With *R*_0_ set to 1.4, 1.7 and 2.0, EP delayed median of 23, 10 and 6 days, respectively
					As above except 99% restriction	With *R*_0_ set to 1.4, 1.7 and 2.0, EP delayed median of 39, 25 and 17 days, respectively
Colizza et al. (2007)^15^	Air travel, global	Mathematical stochastic metapopulation compartmental^b^	A(H5N1)	1.9	20% or 50% air traveller reduction at each connection	No significant impact on EP
Cooper et al. (2006)^16^	Air travel, global	Mathematical stochastic metapopulation model^a^	Epidemic and pandemic influenza	1.8^d^	100% susceptible, 50% air travel reduction, after first 100 symptomatic cases in each city or after 1000 cases in city of origin	EP delayed median of 7 days
				3^d^	40% susceptible, 90% reduction	EP delayed median of 79 days
					As above except 99% reduction	EP delayed median of 131 days
					As above except 99.9% reduction	EP delayed median of 24 days
					100% susceptible, 90% reduction	EP delayed median of 16 days
					As above except 99% reduction	EP delayed median of 30 days
					As above except 99.9% reduction	EP delayed median of 48 days
				5^d^	100% susceptible, 90% reduction	EP delayed median of 13 days
					As above except 99% reduction	EP delayed median of 23 days
					As above except 99.9% reduction	EP delayed median of 35 days
Department of Health (2011)^20^	Evidence-based review	Literature review	Pandemic influenza	NS	90% air travel restriction	ES delayed 1–2 weeks
					99% air travel restriction	ES delayed 2 months
Department of Health (2012)^21^	Modelling summary	Literature review	Pandemic influenza	NS	90% restriction of air travel into United Kingdom	Delay pandemic wave: 1–2 weeks
					99% restriction of air travel into United Kingdom	Delay pandemic wave: 2 months
					Air travel to United Kingdom from South-east Asia – the theoretical origin of epidemic – eliminated	90% reduction in entry of infected travellers, EP in United Kingdom delayed 1–2 weeks
					90% restriction in air travel to United Kingdom from all affected countries	Pandemic wave delayed 3–4 weeks
					As above except 99.9% restriction	Pandemic wave delayed 3–4 months
Eichner et al. (2009)^25^	Air and sea travel, Pacific islands	Mathematical model^a^	A(H1N1)pdm09	1.5, 2.25 or 3.0	79% air and sea travel restriction	With *R*_0_ set to 1.5, 2.25 and 3.0, probability of introduction epidemic reduced by < 1– 65%, < 1–34% and < 1–24%, respectively
					As above but 99% restriction	With *R*_0_ set to 1.5, 2.25 and 3.0, probability of introduction epidemic reduced by < 0.1–98%, < 1–95% and < 1–93%, respectively
Epstein et al. (2007)^31^	Air travel, global	Mathematical stochastic metapopulation model modified^a^	Pandemic influenza	1.7	Hong Kong Special Administrative Region as source of epidemic, 95% restriction implemented after 1000 infectious cases	With epidemic beginning on 1 January or 1 July, ES delayed 13.5 days
					As above except Sydney, Australia, as source of epidemic	With epidemic beginning on 1 January and 1 July, ES delayed 27.2 and 6.7 days, respectively
					As above except London, United Kingdom, as source of epidemic	With epidemic beginning on 1 January or 1 July, ES delayed 0 days
Ferguson et al. (2006)^28^	Internal air, plus border controls, England, Scotland and Wales in United Kingdom and USA	Stochastic mathematical individual-based model^a^	Novel pandemic influenza strain	1.7	90% restriction on entry of infected individuals	IOE delayed 9 days in (England, Scotland and Wales in United Kingdom) or 15 days (USA)
					As above except 99% restriction	IOE delayed 25 days (England, Scotland and Wales in United Kingdom) or 29 days (USA)
					As above except 99.9% restriction	IOE delayed 38 days (England, Scotland and Wales in United Kingdom) or 48 days (USA)
				2.0	90% restriction on entry of infected individuals	IOE delayed 10 days
					As above except 99% restriction	IOE delayed 26 days (England, Scotland and Wales in United Kingdom) or 24 days (USA)
					As above except 99.9% restriction	IOE delayed 40 days (England, Scotland and Wales in United Kingdom) or 43 days (USA)
Flahault et al. (2006)^18^	Air travel, 55 cities worldwide	Mathematical deterministic model^a^	1968–1969-like pandemic influenza	NS	50% travel restriction, at the start of the pandemic or, city-by-city, when there is more than one infectious case per 100 000 population	ES delayed 9 days
Hollingsworth et al. (2006)^33^	Air travel, global	Mathematical stochastic model^a^	H1N1 pandemic influenza	NS	80% air travel restriction, implemented when incidence reaches 100 cases per day	Export of cases delayed 6.6 days
					As above except 90% restriction	Export of cases delayed 13 days
					As above except 99% restriction	Export of cases delayed 133 days
Lam et al. (2011)^14^	International air travel, Hong Kong Special Administrative Region	Mathematical deterministic and stochastic models	Pandemic influenza	1.2, 1.6 or 2.0	Selective air travel restrictions by age, with total ban of air travel by children, implemented 50 days after pandemic starts	With *R*_0_ set to 1.2, 1,6 and 2.0, pandemic arrival delayed: 19–35, < 15 and < 15 days, respectively
Lee et al. (2009)^7^	Systematic review	Deterministic and stochastic models	Various strains of pandemic influenza	1.7–2.0	90% internal and international air travel restrictions	ES delayed 2–3 weeks
				NS	99.9% air travel restriction	National epidemics delayed up to 4 months
				2.4	> 90% restriction of air travel to and from USA	No impact observed
Scalia Tomba and Wallinga (2008)^24^	Border controls, NS	Mathematical deterministic model^c^	Pandemic influenza	2	90% reduction of importation of cases	ES delayed a mean of 11.5 days
					99% reduction of importation of cases	ES delayed a mean of 23 days
					99.9% reduction of importation of cases	ES delayed a mean of 35 days

EP: epidemic peak; ES: epidemic spread; IOE: introduction of epidemic; NS: not specified; *R*_0_: basic reproductive number.

^a^ A so-called SEIR model in which individuals who are susceptible (S), exposed (E), infectious (I) or recovered (R) are considered.

^b^ A so-called SLIR model in which individuals who are susceptible (S), latent (L), infected (I) or permanently recovered (R) are considered.

^c^ Poisson model.

^d^ Maximum value of *R*_0_ modelled.

**Table 6:** Measurement of impact of international travel restrictions on attack rate, cumulative incidence, influenza-like illness peak (i.e. number of cases) and on the number of cases of influenza epidemics

Study	Type of restrictions and setting	Study design	Influenza strain involved	Strain transmissibility (*R*_0_)	Scenario and duration of intervention	Effect estimate
Chong and Ying Zee (2012)^34^	Air, land and sea, Hong Kong Special Administrative Region	Mathematical stochastic model^a^	A(H1N1) pdm2009	1.1, 1.4 or 1.7	90% air travel restriction	With *R*_0_ set to 1.1. 1.4 and 1.7, CINC_7_ was 18%, 50% and 72% of NIV, respectively
					99% air travel restriction	With *R*_0_ set to 1.1. 1.4 and 1.7, CINC_7_ was 18%, 49% and 72% of NIV, respectively
					90% sea travel restriction	With *R*_0_ set to 1.1. 1.4 and 1.7, CINC_7_ was 15%, 55% and 73% of NIV, respectively
					99% sea travel restriction	With *R*_0_ set to 1.1. 1.4 and 1.7, CINC_7_ was 13%, 54% and 73% of NIV, respectively
					90% land travel restriction	With *R*_0_ set to 1.1. 1.4 and 1.7, CINC_7_ was 8%, 51% and 71% of NIV, respectively
					99% land travel restriction	With *R*_0_ set to 1.1. 1.4 and 1.7, CINC_7_ was 5%, 46% and 71% of NIV, respectively
					90% air and sea travel restriction	With *R*_0_ set to 1.1. 1.4 and 1.7, CINC_7_ was 18%, 48% and 70% of non-intervention value, respectively
					99% air and sea travel restriction	With *R*_0_ set to 1.1. 1.4 and 1.7, CINC_7_ was 16%, 45% and 70% of NIV, respectively
					90% air and land travel restriction	With *R*_0_ set to 1.1. 1.4 and 1.7, CINC_7_ was 15%, 40% and 71% of NIV, respectively
					99% air and land travel restriction	With *R*_0_ set to 1.1. 1.4 and 1.7, CINC_7_ was 5%, 35% and 70% of NIV, respectively
					90% land and sea travel restriction	With *R*_0_ set to 1.1. 1.4 and 1.7, CINC_7_ was 15%, 50% and 72% of NIV, respectively
					99% land and sea travel restriction	With *R*_0_ set to 1.1. 1.4 and 1.7, CINC_7_ was 13%, 48% and 72% of NIV, respectively
					90% air, land and sea travel restriction	With *R*_0_ set to 1.1. 1.4 and 1.7, CINC_7_ was 3%, 28% and 68% of NIV, respectively
					99% air, land and sea travel restriction	With *R*_0_ set to 1.1. 1.4 and 1.7, CINC_7_ was < 1%, < 5% and 25% of NIV, respectively
Ciofi degli Atti et al. (2008)^17^	Air travel, Italy	Mathematical deterministic metapopulation^a^ and individual-based model	NS	1.4, 1.7 or 2.0	90% air travel restriction, implemented from 30 days after record of first case for the whole pandemic until 2 months after introduction of first case in Italy	With *R*_0_ set to 1.4. 1.7 and 2.0, CAR was 21.2%, 30.8% and 38.7% of NIV and PDAR was 0.42%, 1.01% and 1.90% of NIV, respectively
					As above except 99% air travel restriction	With *R*_0_ set to 1.4. 1.7 and 2.0, CAR was 21.1%, 30.8% and 38.7% of NIV and PDAR was 0.40%, 1.03% and 1.91% of NIV, respectively
Colizza et al. (2007)^15^	Air travel, global	Mathematical stochastic metapopulation model^b^	A(H5N1)	1.9	20% or 50% air travel restriction	No impact on CAR
Epstein et al. (2007)^31^	Air travel, global	Mathematical stochastic metapopulation model^c^	Pandemic influenza	1.7	Hong Kong Special Administrative Region as source of epidemic, 95% restrictions implemented after 1000 infectious cases	If epidemic begins on 1 January or 1 July, it produces global means of 81 531 156 and 132 230 576 cases, respectively
					As above except Sydney, Australia, as source of epidemic	If epidemic begins on 1 January or 1 July, it produces global means of 33 068 217 and 94 823 730 cases, respectively
					As above except London, United Kingdom, as source of epidemic	If epidemic begins on 1 January or 1 July, it produces global means of 118 523 844 and 7 134 433 cases, respectively
Kernéis et al. (2008)^19^	Air travel, 52 cities worldwide	Mathematical stochastic metapopulation deterministic model^a^	Pandemic influenza strain (NS)	1.8 or 4.9	Air travel restrictions of unspecified effectiveness, over various, unspecified timelines	Little effect on global burden or spatial and temporal diffusion of influenza pandemic
Lee et al. (2009)^7^	Several scenarios	Systematic review (deterministic and stochastic models)	Pandemic influenza (different strains)	1.7 or 2.0	90%, 99% or 99.9% air travel restriction	With *R*_0_ set to 1.7 and 2.0 there was, respectively, no impact on overall attack rate and a 1% increase in that rate – with a 20% increase in PDAR
Marcelino and Kaiser (2012)^32^	Air travel, 500 major airports, worldwide	Mathematical stochastic metapopulation model^a^	A(H1N1)pdm09	1.7	Cancellation of a quarter of flight connections between 500 cities	Number of circulating infected individuals reduced by an additional 19%

CAR: cumulative attack rate; CINC_7_: cumulative incidence seven months after start of epidemic; NIV: non-intervention value; NS: not specified; PDAR: peak daily attack rate; *R*_0_: basic reproductive number.

^a^ A so-called SEIR model in which individuals who are susceptible (S), exposed (E), infectious (I) or recovered (R) are considered.

^b^ A so-called SLIR model in which individuals who are susceptible (S), latent (L), infected (I) or permanently recovered (R) are considered.

^c^ The model took into account individuals who were nonsusceptible (NS), susceptible (S), exposed (E), infectious (I) or recovered (R).

In Italy, relatively large delays were reported in reaching an influenza A(H5N1) peak – i.e. 7–37 days, depending on the level of influenza transmissibility and the extent of the restrictions simulated.^17^ Travel restrictions had no beneficial effect on attack rate if the level of strain transmissibility was moderate or high.^17^ In a more recent review, it was estimated that introduction of pandemic influenza into the United Kingdom could be delayed by up to 2 months if there was an almost complete – e.g. 99.9% – ban on air travel.^20^ However, the size of the effect was considerably reduced, to just 1–2 weeks, if the level of restriction was lowered to 90%.^20^ Similar observations were made in an assessment of the impact of restrictions of air, land and sea travel on the introduction of H1N1 pdm09 into Hong Kong Special Administrative Region (SAR), China.^34^ In this study, it was estimated that restrictions of 90% and 99% on all modes of transportation would delay the epidemic peak by up to 6 and 12 weeks, respectively, when *R*_0_ was set to 1.4.^34^ When *R*_0_ was set to 1.7, a restriction of 99% on all modes of transportation would delay the epidemic peak by up to 8 weeks and halve the cumulative attack rate. Air travel restrictions appeared to be the most effective isolated intervention, even though most infected cases would probably enter Hong Kong SAR by land travel from mainland China.^34^ Although one review of the evidence from mathematical modelling concluded that air travel bans would probably have a similar effect irrespective of the pandemic’s country of origin,^21^ another report believed that the effectiveness of such restrictions would vary according to the geographical source of the pandemic.^31^ If air travel bans delayed the epidemic so that it coincided with the usual influenza season, the apparent number of cases and the size of the peak in the epidemic could both increase.^31^ However, the opposite trends might be observed if the travel restrictions coincided with a period of low strain transmissibility.^31^ By restricting air travel by 95%, it should be possible to delay pandemic spread across the USA – of an infection originating in Sydney or Hong Kong SAR – by 2–3 weeks.^31^ However, there was no corresponding impact if the geographical origin of the pandemic was London because of London’s high flight densities and interconnectivity.^31^ The selective cancellation of a quarter of all connection flights between 500 major cities worldwide could be more effective than the closure of all of the cities’ airports – reducing the number of infected travellers by an additional 19%.^32^ A review of air travel restrictions between Asia and the United Kingdom indicated that such restrictions would stop no more than 90% of infected travellers from the pandemic’s country of origin.^21^ If air travel from all affected countries was restricted by 90.0% and 99.9%, the pandemic wave would be delayed by 3–4 weeks and up to 4 months, respectively,^21^^,^^28^ but such intensive restrictions would clearly have negative social and economic impacts. A systematic review found that extensive air travel restrictions – e.g. restrictions of more than 90% – could delay the spread of pandemics by up to 4 months if the strains involved had low to moderate transmissibility.^7^ However, such restrictions appeared ineffective if the strains involved had high transmissibility – i.e. if *R*_0_ was 2.4.^7^ In general, a combination of interventions appeared to be more effective than the implementation of travel restrictions in isolation.^7^

## Discussion

The results of our systematic review indicate that overall travel restrictions have only limited effectiveness in the prevention of influenza spread, particularly in those high transmissibility scenarios in which *R*_0_ is at least 1.9 (Box 2). The effect size varied according to the extent and timeliness of the restrictions, the size of the epidemic, strain transmissibility, the heterogeneity of the travel patterns, the geographical source and the urban density of international travel hubs. Only extensive travel restrictions – i.e. over 90% – had any meaningful effect on reducing the magnitude of epidemics. In isolation, travel restrictions might delay the spread and peak of pandemics by a few weeks or months but we found no evidence that they would contain influenza within a defined geographical area.

Box 2 **Summary of findings of the 23 studies assessed**

**Internal travel restrictions: general observations**

- Have limited effectiveness
- Delay pandemic spread by about 1 week
- Delay pandemic peak by about 1.5 weeks
- Have little impact on magnitude of pandemics – e.g. they may reduce attack rates by < 2%
- Simulated impact is particularly weak in scenarios that involve strains with high transmissibility

**Internal travel restrictions: risk of bias assessment**

- Relevant studies have low to moderate risk of bias
- Paucity of data on terrestrial travel may have led to an overestimation of the impact of travel restrictions
- Many simulations take no account of the characteristics of human populations – e.g. the mixing and variation of susceptibility across age groups – or of seasonality. Such limitations could well have affected the simulated spread of pandemic waves and impacts of interventions

**International travel restrictions: general observations**

- Have limited effectiveness – e.g. 90% air travel restriction in all affected countries may delay spread of pandemics by 3–4 weeks
- Have minimal impact on the magnitude of pandemics, typically reducing attack rates by less than 0.02%
- May prolong the seasonal influenza season
- May result in higher epidemic peak if resultant delay causes pandemic wave to coincide with seasonal influenza wave
- Simulated impact particularly weak in scenarios that involve strains with high transmissibility
- Extensive restriction of international air travel might delay introduction of a pandemic into a country by up to 2 months and delay pandemic spread by 3–4 months
- Would not prevent introduction of a pandemic into any given country
- May give time for other interventions – e.g. the production and distribution of effective vaccines and antiviral drugs
- Social and economic impacts need to be evaluated

**International travel restrictions: specific measures**

- May have benefits compared with more widespread restrictions – e.g. in one simulation, compared with the closure of all of the cities’ airports, the targeted reduction of a quarter of flight connections between 500 major cities gave a greater reduction in the number of infected travellers
- Compared with banning air travel by adults, the banning of air travel by children may be more effective at delaying the spread of a pandemic but is socially impractical

**International travel restrictions: risk of bias assessment**

- Relevant studies have low to moderate risk of bias
- A paucity of data on travel by sea and land may have led to an overestimation of the impact of air travel restrictions on the containment of influenza pandemics
- Much of the information available on air travel has a lack of detail on flight destinations and numbers of travellers and this may have led to inaccurate assumptions being made about the spread of influenza
- Again, many simulations take no account of the characteristics of human populations – e.g. the mixing and variation of susceptibility across age groups – or of seasonality and such limitations could well have affected the simulated spread of pandemic waves and impacts of interventions
- When simulating novel pandemic strains, validation of models was an issue; mathematical models need to be validated against surveillance data to improve their value as predictive tools for policy-makers

Several limitations associated with our review warrant discussion. We included mathematical modelling studies that simulated very diverse scenarios with varying levels of *R*_0_, geographical locations, means of transportation, strains and population characteristics. A paucity of surveillance data concerning the impact and effectiveness of nonpharmaceutical interventions meant that our observations had to be mainly based on simulations.^6^ While mathematical models are important tools that can be used to inform policy-makers, they cannot account fully for all aspects of real-life situations.

The lack of available data from observational or experimental studies precluded the conduct of the meta-analysis and sensitivity analysis that formed part of the protocol that we registered.^8^ Most of the studies that we included in our review used probabilistic models that appeared to have adequate levels of complexity to simulate disease spread and the impact of interventions. In comparison, deterministic models are less complex and do not take uncertainty into account but are still useful when limited data are available and a rapid simulation is needed.^7^ Most of the studies we reviewed were limited by a lack of consideration of heterogeneous mixing, socioeconomic status and the relationship between age and immunity.^37^ Many also simulated constant strain transmissibility during epidemics – even though transmissibility can vary over time because of seasonal climactic conditions, changes in host susceptibility and the effects of interventions such as social distancing, quarantine and the use of antiviral drugs.^38^ The authors of some of the articles noted concerns that may have affected model accuracy, such as issues with the quality of air travel data – e.g. a lack of flight itineraries^28^ – and the need to use crude estimates of the volume of travellers within and between countries. There was a general paucity of data on land and sea travel,^25^ although one of the studies provided comprehensive data on such travel.^34^ The tool we developed to assess the risk of bias in the mathematical modelling studies has not been validated and could have produced imprecise estimates.

The results of several studies indicate that, in reducing the global spread of influenza and the overall number of infected individuals, a combination of several different interventions is more effective than any single isolated measure.^16^^,^^17^^,^^34^ One study estimated that, when the strains involved have moderate transmissibility, a combination of antiviral prophylaxis, extensive travel restrictions and infant vaccination could reduce the cumulative attack rate by 77–87%.^17^ However, effective vaccines are not generally available at the point of emergence of a novel pandemic virus. The effectiveness of combined or single interventions can be affected by the timeliness of the implementation^4^^,^^39^ and this appears to be particularly relevant with strains of higher transmissibility.^34^

Often, in the context of pandemic preparedness and response, travel restrictions – especially at points of entry – have intuitive appeal to policy-makers because they demonstrate that a tangible attempt is being made to prevent the ingress of a novel virus or prevent onward spread. However, such an attempt is not always effective. *WHO interim protocol: rapid operations to contain the initial emergence of pandemic influenza* is implicitly focused on the creation of geographical cordons within a country and places more emphasis on the restriction of travel by land than on restrictions of air or sea travel.^1^ However, the relevant data that are available seem to indicate that restrictions on land travel would have a limited impact on containment or even on the slowing of transmission.^34^

It seems likely that, for delaying the spread and reducing the magnitude of an epidemic in a given geographical area,^7^ a combination of interventions would be more effective than isolated interventions.^16^^,^^34^ Travel restrictions per se would not be sufficient to achieve containment in a given geographical area, and their contribution to any policy of rapid containment is likely to be limited.
